# Multifunctional Silver Nanoparticles Based on Chitosan: Antibacterial, Antibiofilm, Antifungal, Antioxidant, and Wound-Healing Activities

**DOI:** 10.3390/jof8060612

**Published:** 2022-06-08

**Authors:** Amr M. Shehabeldine, Salem S. Salem, Omar M. Ali, Kamel A. Abd-Elsalam, Fathy M. Elkady, Amr H. Hashem

**Affiliations:** 1 Botany and Microbiology Department, Faculty of Science, Al-Azhar University, Nasr City, Cairo 11884, Egypt; dramrshehab@azhar.edu.eg; 2 Department of Chemistry, Turabah University College, Turabah Branch, Taif University, Taif 21944, Saudi Arabia; om.ali@tu.edu.sa; 3 Plant Pathology Research Institute, Agricultural Research Centre, Giza 12619, Egypt; kamelabdelsalam@gmail.com; 4 Microbiology and Immunology Department, Faculty of Pharmacy (Boys), Al-Azhar University, Nasr City, Cairo 11884, Egypt; fathyelkady2426.el@azhar.edu.eg

**Keywords:** chitosan, chitosan/silver nanoparticles, antimicrobial, antioxidant, wound healing

## Abstract

The purpose of this study is to create chitosan-stabilized silver nanoparticles (Chi/Ag-NPs) and determine whether they were cytotoxic and also to determine their characteristic antibacterial, antibiofilm, and wound healing activities. Recently, the development of an efficient and environmentally friendly method for synthesizing metal nanoparticles based on polysaccharides has attracted a lot of interest in the field of nanotechnology. Colloidal Chi/Ag-NPs are prepared by chemical reduction of silver ions in the presence of Chi, giving Chi/Ag-NPs. Physiochemical properties are determined by Fourier-transform infrared spectroscopy (FTIR), X-ray diffraction (XRD), transmission electron microscopy (TEM), dynamic light scattering (DLS), and scanning electron microscopy with energy-dispersive X-ray spectroscopy (SEM-EDX) analyses. TEM pictures indicate that the generated Chi/Ag-NPs are nearly spherical in shape with a thin chitosan covering around the Ag core and had sizes in the range of 9–65 nm. In vitro antibacterial activity was evaluated against *Staphylococcus aureus* and *Pseudomonas aeruginosa* by a resazurin-mediated microtiter plate assay. The highest activity was observed with the lowest concentration of Chi/Ag-NPs, which was 12.5 µg/mL for both bacterial strains. Additionally, Chi/Ag-NPs showed promising antifungal features against *Candida albicans*, *Aspergillus fumigatus*, *Aspergillus terreus,* and *Aspergillus niger*, where inhibition zones were 22, 29, 20, and 17 mm, respectively. Likewise, Chi/Ag-NPs revealed potential antioxidant activity is 92, 90, and 75% at concentrations of 4000, 2000, and 1000 µg/mL, where the IC_50_ of Chi/Ag-NPs was 261 µg/mL. Wound healing results illustrated that fibroblasts advanced toward the opening to close the scratch wound by roughly 50.5% after a 24-h exposure to Chi/Ag-NPs, greatly accelerating the wound healing process. In conclusion, a nanocomposite based on AgNPs and chitosan was successfully prepared and exhibited antibacterial, antibiofilm, antifungal, antioxidant, and wound healing activities that can be used in the medical field.

## 1. Introduction

Antimicrobial resistance occurs when bacteria, viruses, fungi, and parasites evolve over time and become less drug-responsive, making infections more difficult to treat and raising the risk of sickness, severe illness, and death. Antibiotics and other antimicrobial drugs are rendered ineffective by drug resistance, and infections are becoming increasingly difficult or impossible to treat. Therefore, it is necessary to design and develop new compounds that overcome these limitations. Recently, nanoparticles (NPs) have been successfully used to deliver therapeutic agents [1,2,3,4] in the diagnosis of chronic diseases, to reduce bacterial infections, and in the food and clothing industries as antimicrobial agents [5,6,7,8,9]. Because of their antimicrobial properties and unique mode of action, specialized NPs provide an attractive alternative to conventional antibiotics in the development of a new generation of antibiotics. Green synthesis of metallic NPs is an economical, easy, and environmentally friendly approach [10,11,12,13,14,15,16,17]. Due to their various applications in the medical field, silver NPs (AgNPs) have been proposed as treatment agents to overcome the problem of drug resistance caused by the abuse of antibiotics [18,19,20]. The mechanism by which silver exerts its biological activity is still poorly understood. The bacteria that makeup biofilms are a primary concern of medical microbiology. These membranes are formed by secreting a layer of polymer consisting of sugars, nucleic acids, and proteins, which are formed on biological and non-biological membranes and surfaces [21,22]. This layer plays an important role by restricting the diffusion of antibiotics into biofilm-producing cells and making them resistant to antibiotics. It has been found that bacterial cells growing within biofilms secrete different surface molecules and virulence factors and exhibit low growth rates, which enhance their pathogenicity by several folds [23]. Chitosan is a linear polysaccharide that is obtained from the deacetylation of chitin, a naturally occurring polymer present in the shells of prawns and other crustaceans [24]. It is one of the most commonly used biopolymers in a wide range of applications, including fabrics, cosmetics, water treatment, and food processing [25,26,27]. Previous studies confirmed that chitosan has multiple roles in nanoparticle synthesis, stabilization, and their applications [28,29]. The wound healing process is described by a series of responses including inflammatory, tissue regenerating, and tissue-remodeling processes [30]. Wound dressings are biomaterials of synthetic or natural origin that support wound healing by providing suitable micro-environments capable of attracting the cells to the wounded area [31]. In recent years, among the various wound dressing options, hydrogels have been highlighted for their unique properties, thus making them ideal for promoting an environment conducive to tissue regeneration [32]. Herein, AgNPs have been synthesized in the presence of chitosan using a new environmentally friendly synthesis process, and their biological activities, namely, antibacterial, antibiofilm, antifungal, antioxidant, and wound healing promoting skills, have been evidenced.

## 2. Materials and Methods

### 2.1. Synthesis of Chitosan/Silver Nanoparticles (Chi/Ag-NPs)

Chitosan/silver nanoparticles (Chi/Ag-NPs) were synthesized utilizing a chemical reduction process with chitosan as a reducing and stabilizing agent. With minor changes, the synthesis of Chitosan/Silver nanoparticles (Chi/Ag-NPs) was carried out according to the technique reported by [33]. Chitosan (0.2%) was produced by mixing in 0.5% acetic acid. After that, the chitosan solution was filtered to create a homogeneous solution. NaOH and AgNO_3_ had just been made. To the chitosan solution, an aliquot of 2 mL of 2 mM AgNO_3_ and 100 µL of 0.5 M NaOH (pH 10) was added. At 85 °C, the chitosan solution was agitated for 4 h. The colorless chitosan solution became yellow, then brown, indicating that Chi/Ag-NPs had been synthesized. Finally, the Chi/Ag-NPs solution was stored, and the precipitate was filtered, washed with distilled water, and dried in an oven at 90 °C for 4 h. Chitosan purchased from Sigma-Aldrich (St. Louis, MO, USA), and Silver nitrate (AgNO_3_) was obtained from Fisher Scientific (Mumbai, India). Other chemicals and reagents used in this study were purchased from Modern Lab Co., Madhya Pradesh, India, in analytical grade without any purification required.

### 2.2. Characterization of Chi/Ag-NPs

A variety of instrumental analytical methods were used to characterize the Chi/Ag-NPs. Using a Spectrum Two IR Spectrometer (PerkinElmer Inc., Shelton, WA, USA) and these techniques, the total internal reflectance/Fourier-transform Infrared (ATR-FTIR) spectra was used to semi-quantitatively measure the observable IR spectrum of the Chi/Ag-NPs by evaluating the transmittance over a spectral region of 4000 to 400 cm^−1^. To achieve a suitable signal quality, all spectra were collected at a 4 cm^−1^ resolution by collecting 32 scans [34]. A Diano X-ray diffractometer (Philips) with a CuK radiation source (λ = 0.15418 nm) activated at 45 kV, as well as a generator (PW, 1930) and a goniometer (PW, 1820), was used to study the XRD pattern of the produced Chi/Ag-NPs. The shape and size of the prepared Chi/Ag-NPs were observed using the TEM method. The Ultra-High Resolution transmission electron microscope (JEOL-2010, Tokyo, Japan) with a voltage of 200 kV was employed. A drop of the particle solution was placed on a carbon-coated copper grid and dried under a light to create TEM grids. The NicompTM-380 ZLS size analyzer from the United States (USA) was used to calculate the pore size distribution and zeta potential of the prepared Chi/Ag-NPs using dynamic light scattering (DLS). For particle size detection, laser beam scattering at 170° was utilized, with the zeta potential recorded at 18°. A field emission scanning electron microscope (SEM) installed with a Field Emission-Gun (Quanta, 250-FEG) and connected with an energy-dispersive X-ray analyzer (EDX, Unit) with an excitation source of 30 kV for energy-dispersive X-ray evaluation (EDX) and mapping were used to examine the surfaces of the prepared Chi/Ag-NPs.

### 2.3. Microbial Strains and Reagent

*Staphylococcus aureus* ATCC^®^ 25923™ and *Pseudomonas aeruginosa* MTCC1034 were cultivated in Luria broth medium and incubated at 37 °C, for 16–18 h. Fungal strains used are *Candida albicans* ATCC 90028, *Aspergillus niger* RCMB 02724, *A. terreus* RCMB 02574, and *A. fumigatus* RCMB 02568. These four fungal strains were inoculated on malt extract agar (MEA) (Oxoid) plates; then incubated for 3–5 days at 28 ± 2 °C; then kept at 4 °C for further use [35,36,37,38]. Chitosan, low molecular weight, and crystal violet were purchased from Sigma-Aldrich (St Louis, MO, USA). MTT [3-(4,5-dimethylt hiazol-2-yl)-2,5-diphenyltetrazolium bromide]. Silver nitrate (AgNO3) was obtained from Fisher Scientific (Mumbai, India). Normal human skin cell line (BJ-1) was cultured in RPMI 1640 medium supplemented with 10% heated fetal bovine serum, 1% of 2 mM l-glutamine, 50 IU/mL penicillin, and 50 µg/mL streptomycin.

### 2.4. Broth Microdilution Assay

Briefly, a fresh culture on LB broth media at a turbidity equivalent to that of 0.5 McFarland standard, 500 μL of each bacterial culture were added into a 96-well polystyrene flat-bottomed microtiter plate [39]. Tested sample was added to bacterial suspension in each well at a final concentration ranging from 0 to 1000 µg/mL. Growth control wells contained only bacteria in LB media. Two-fold serial dilutions of the tested samples Chi/Ag-NPs, were made starting with the first well by adding 50 μL of the tested sample, dissolved at a concentration of 1000 µg/mL. To each of the wells, 10 μL of the diluted culture (0.5 McFarland standards) was added. After incubation at 37 °C for 24 h, 5 μL resazurin indicator (prepared by dissolving 0.016 g in 100 mL of sterile distilled water) was also added to all 96 wells. Then the microtiter plate was incubated in the dark. We observed with the naked eye any change in color from purple to pink as a positive result. The lowest concentration of the tested sample in which discoloration occurred was recorded as the MIC value. All experiments were performed in triplicate [40]. The MBC for each sample was calculated by plating the contents of the first three wells that showed no visible bacteria growth onto LB plates and incubating for 24 h [41].

### 2.5. Evaluation of Anti-Biofilm Activity

Biofilm experiments were performed using static biofilm model. Effect of tested sample Chi/Ag-NPs on *S. aureus* ATCC^®^ 25923™ and *P. aeruginosa* MTCC1034 biofilm formation were determined by the crystal violet staining method [42,43]. Prepared compound was diluted into 96-well plates as described above; six concentrations diluted from 0.5xMIC. The plates were incubated under aerobic conditions at 37 °C for 48 h. discarding the liquid mixture; the wells were stained with 0.1 mL 0.4% crystal violet for 15 min after being washed with sterile water twice. Then, samples were rinsed with distilled water twice and the dye bound to biofilm was solubilized by adding ethanol (95%). Absorbance of the isolated dye was measured quantitatively at 540 nm. The biofilm inhibition percentage was calculated using the following formula [44]:

[(OD growth control − OD sample)/OD growth control] × 100

OD: optical density

### 2.6. Antifungal Activity

The test of diffusion in agar was performed in accordance with the document M51-A2 of the Clinical Laboratory Standard Institute [45] with minor adaptations. Fungal strains were initially grown on MEA plates and incubated at 30 °C for 3–5 days [46,47,48]. The fungal suspension was prepared in sterilized phosphate buffer solution (PBS) pH 7.0, and then the inoculum was adjusted to 10^7^ spores/mL after counting in a cell counter chamber. One ml was uniformly distributed on agar MEA Plates. In total, 100 µL of each Chi/Ag-NPs, Ag+, Chi, and nystatin were put in Agar wells (7 mm) then incubated at 30 °C. After 72 h of incubation, the inhibition zone diameter was measured [49,50].

### 2.7. Antioxidant Activity

Different concentrations Chi/Ag-NPs, Ag+, Chi, and Ascorbic acid rangeing from 3.9 to 4000 µg/mL were used to determine the ability to scavenge DPPH (2,2-diphenyl-1-picrylhydrazyl) radicals. DPPH solution (800 µL) was mixed with 200 μL of the specific concentration and incubated for 30 min at 25 °C in darkness. After this time, centrifugation was performed at 13,000 rpm for 5 min, then absorbance was measured at 517 nm [46]. Antioxidant activity was calculated by the following equation:

$$\mathrm{Antioxidant}\;\mathrm{activity}\;\left(\%\right)=\frac{\mathrm{Control}\;\mathrm{absorbance}-\mathrm{Sample}\;\mathrm{absorbance}}{\mathrm{Control}\;\mathrm{absorbance}}\times 100$$

### 2.8. Determination of Safe Dose on the Proliferation of Normal Human Skin Fibroblast Cell Line by Sulphorhodamine B (SRB) Assay

Cytotoxicity was investigated using normal human skin cell line (BJ-1), Cell viability was assessed by SRB assay [51]. Aliquots of 100 μL cell suspension (5 × 10^3^ cells) were in 96-well plates and incubated in complete media for 24 h. Before addition to the culture medium, tested substance Chi/Ag-NPs and standard substance drugs doxorubicin (DOX) were dissolved in DMSO and followed by serial dilution for 6 points ranging from 200 µg/mL to 1.56 µg/mL. After 72 h of exposure, cells were fixed by replacing media with 150 μL of 10% TCA and incubated at 4 °C for 1 h. Aliquots of 70 μL SRB solution (0.4% *w*/*v*) were added and incubated in a dark place at room temperature for 10 min. Plates were washed 3 times with 1% acetic acid and allowed to air-dry overnight. Then, 150 μL of TRIS (10 mM) was added to dissolve protein-bound SRB stain [52]. The effective safe concentration (EC100) value (at 100% cell viability) of each tested extract was estimated by GraphPad Instat software (version 6.01), California, USA. GraphPad Instat software was used to evaluate the cytotoxicity activity of the tested material, which was expressed as an IC_50_ value. The experiments were carried out three times. As a positive control, doxorubicin was used. The levels of cytotoxic effects were categorized as cytotoxic (IC_50_ 2.00 µg/mL), moderately cytotoxic (IC_50_ 2.00–89.00 µg/mL), and non-toxic (IC_50_ > 90.00 µg/mL) according to the Special Programme for Research and Training in Tropical Diseases (WHO—Tropical Diseases) [53]. The rate of the cytotoxicity (CT%) was estimated by the following expression:

CT% = Ac − At/Ac × 100

In which, Ac and At are the absorbance of the control sample and the test sample, respectively.

### 2.9. In Vitro Wound-Healing Assay

The wound-healing potential of the final formulations was assessed by in vitro wound-healing assay [54]. To this aim, Human Skin Fibroblast cell line were seeded at a density of 3 × 10^5^/well onto a coated 6-well plate in 5% FBS-DMEM at 37 °C and 5% CO_2_ to obtain a monolayer of cells [55] then, a scratch was made across the middle of each well using a sterile 1000 μL pipet tip. The plate was washed thoroughly with PBS. Control wells were replenished with fresh medium while drug wells were treated with fresh media containing drug. Images were taken using an inverted microscope at the indicated time intervals. The plate was incubated at 37 °C and 5% CO_2_ in-between time points. The migration rate can be expressed as the percentage of area reduction of wound closure, which increases as cells migrate over time [56].

Wound closure%: (A_0_ − A_t_/A_0_) × 100,

where A_0_ = 0 h is the average area of the wound measured immediately after scratching (time zero), and A_t_ = Δh is the average area of the wound measured h hours after the scratch is performed.

### 2.10. Statistical Analysis

Data are presented as means ± SD of at least three independent experiments. Comparisons are made by the Student’s *t*-test or by ANOVA when appropriate. Differences are considered statistically significant at *p* < 0.05. Statistical analysis was carried out estimated by GraphPad Instat software, (version 6.01), San Diego, CA, USA.

## 3. Results and Discussion

### 3.1. Characterization of Chi/Ag-NPs

**Figure 1:**
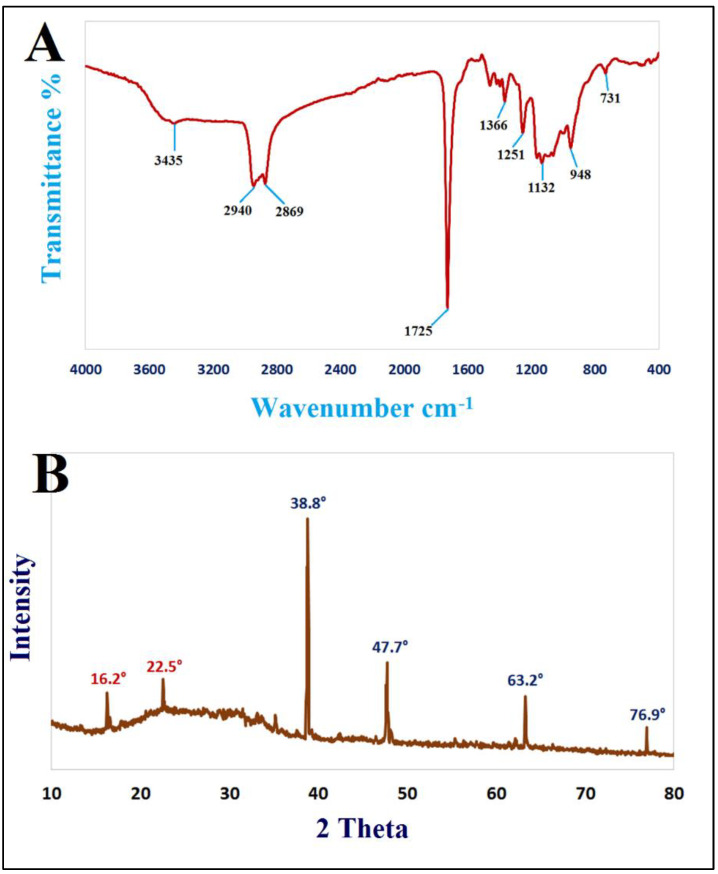
FTIR spectrum (**A**) and XRD pattern (**B**) of Chi/Ag NPs.

Nanostructures have piqued curiosity as a fast-evolving class of materials with a wide range of uses. Nanomaterials have been described using a number of methodologies to explain their size, crystalline structure, elemental content, and a range of other physical features. There are various physical qualities that may be examined using many methods. The many strengths and limits of each methodology make it difficult to choose the best method, and an aggregate characterization approach is frequently necessary [57]. FTIR analysis of Chi/Ag-NPs was carried out to validate the decrease in molecular interaction and capping agent, which is important for the nanoparticles’ synthesis and stabilization. This method is commonly used to analyze nanostructures qualitatively. The FTIR spectra of Ag NPs revealed absorption peaks at 3435 cm^−1^, 2940 cm^−1^, 2869 cm^−1^, 1725 cm^−1^, 1366 cm^−1^, 1251 cm^−1^, 1132 cm^−1^, 948 cm^−1^, and 731 cm^−1^, which correspond to linkage groups (Figure 1A). Moreover, the peaks at 3435 cm^−1^ matched to -OH-group stretch-vibrations. Asymmetric and symmetric -CH_2_ groups may be ascribed to the bands at 2940 cm^−1^ and 2869 cm^−1^, respectively. Carbonyl expands vibrations in aldehydes, ketones, and carboxylic acids, which correlate to the peak at 1725 cm^−1^. The existence of a strong 1725 cm^−1^ signal in Chi/Ag-NPs shows that silver ion (Ag+) reduction is accompanied by hydroxyl group oxidation in chitosan structures. C-N- stretching is shown by the peak seen at 1366 cm^−1^. The -C–O–C stretching is responsible for the peak at 1251 cm^−1^. The C-O stretching vibration is shown by the peak at 1132 cm^−1^. The absorption peak at 948 cm^−1^ conforms to the β-d-glucose unit’s typical absorption. One of the most extensively used methods for the characterization of NPs is X-ray diffraction (XRD). The crystalline nature, phase behavior, lattice constants, and particle sizes are commonly determined using XRD. The significant peaks appeared at 38.8°, 47.7°, 63.2°, and 76.9°, correlating to lattice-planes (111), (200), (220), and (311) are shown in XRD diffraction of synthesized Chi/Ag-NPs. The Chi/Ag-NPs XRD results were found to be highly similar to JCPDS card no. 04-0783. As a result, XRD revealed that AgNPs were generated by reducing AgNO_3_ with chitosan (Figure 1B), and the crystal lattice complemented previous studies. Furthermore, the most prominent diffraction peaks at 16.2° and 22.5° confirmed the crystalline form of chitosan [58].

**Figure 2:**
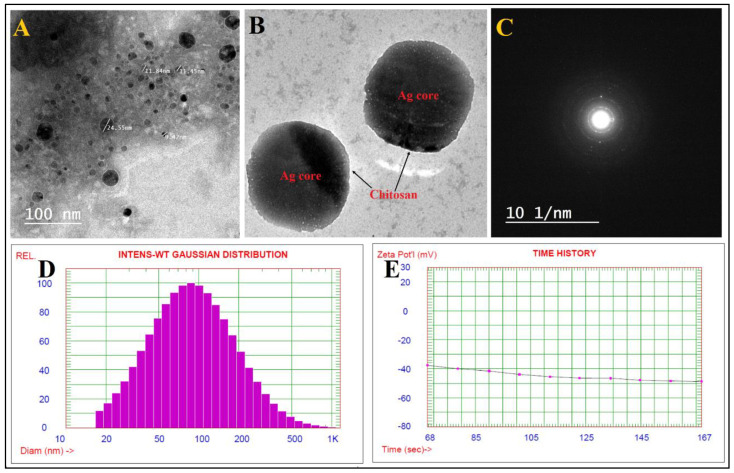
TEM images (**A**,**B**), SAED pattern (**C**), DLS (**D**),and zeta potential (**E**) of Chi-Ag-NPs.

The TEM pictures indicated that the generated Chi/Ag-NPs were nearly spherical, polydisperse, and had sizes in the range of 9–65 nm Figure 2A. The particles had a spherical shape with a thin chitosan covering around the Ag core (Figure 2B). Furthermore, TEM micrographs revealed a uniform dispersion of the chitosan covering surrounding the Ag-nanostructures. When Chi/Ag-NPs were tested, no aggregation was found, indicating that the nanostructures were entirely covered with a polymer. Ag-nanoparticles coated with chitosan did, in fact, have a transparent coating surrounding their core. In another paper, it was discovered that the size of Chi/Ag-NPs ranges around 10–230 nm [33]. Figure 2C shows the Chi/Ag-NPs’ areas selected electron diffraction (SAED) pattern, which shows excellent sharp rings and confirms the Ag-nanostructures’ crystalline character. DLS is among the most often used techniques to detect the distribution of particle size in a colloidal mixture based on intensity. The Chi/Ag-NPs produced were a polydispersed combination of spherical Chi/Ag-NPs with an average diameter of 117.6 nm (Figure 2D). Because the acquired size utilizing DLS is not only connected to the metallic core of particles but also impacted by capping proteins around the particles, the resulting particle sizes were over expressed when compared to those observed utilizing TEM. The Chi/Ag-NPs had a zeta potential of −48.76 mV (Figure 2E), showing that Chi/Ag-NPs in suspension were dispersed optimally. The surface charge of the Chi/Ag-NPs is represented by the negative charge of the zeta potential value.

**Figure 3:**
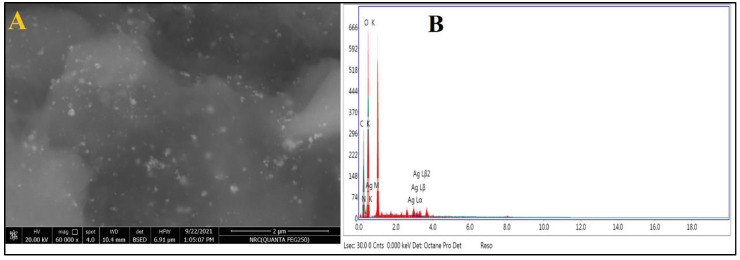
(**A**) SEM image and (**B**) EDX spectrum of prepared Chi/Ag-NPs.

As shown in Figure 3A, the SEM was used to evaluate the surface morphology and particle size of Chi/Ag-NPs. Chi/Ag-NPs had a form that was virtually spherical. The particle size varied from 24 to 80 nm on average. EDX analysis was used to determine the elemental composition of the Chi/Ag-NPs powder. In the Chi/Ag-NPs, the EDX spectra revealed the existence of several well-defined bands associated with silver [Ag], oxygen [O], and carbon [C] components (Figure 3B). The carbon [C] and oxygen [O] signals come from the chitosan, whereas the silver [Ag] peak indicates the creation of Ag-nanostructures. Furthermore, EDX spectra revealed the generation of very pure Chi/Ag-NPs with no additional impurity-related peaks.

### 3.2. Determination of Minimum Inhibitory Concentration (MIC) by Resazurin Stain

**Figure 4:**
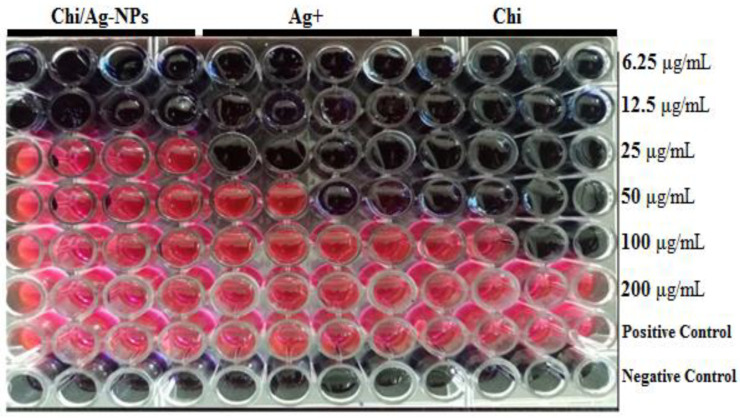
Resazurin dye test for determining minimum inhibitory concentration against *S. aureus* ATCC^®^ 25923™ and *P. aeruginosa* MTCC1034.

In this study, Chi/Ag-NPs were tested as an antimicrobial against selected gram-positive and gram-negative bacteria. Using a resazurin-mediated microtiter plate assay, the antibacterial activity of Chi, Ag+, and Chi/Ag-NPs was investigated against *P. aeruginosa* and *S. auerus* bacterial strains. The color shift of the resazurin indicator was used to visually assess the inhibitory action of Chi, Ag+, and Chi/Ag-NPs. Figure 4 depicts the color shift seen at various concentrations of Chi, Ag+, and Chi/Ag-NPs. Chitosan and Ag+ exhibited moderate antibacterial activity against *P. aeruginosa* and *S. auerus,* with MICs ranging from 25 to 100 µg/mL, while Chi/Ag-NPs had increased antibacterial activity against both bacterial strains. Figure 4 shows the MIC and MBC values for Chi, Ag+, and Chi/Ag-NPs. The highest activity was observed with the lowest concentration of Chi/Ag-NPs, which was 12.5 µg/mL for both *P. aeruginosa and S. auerus*. Table 1 summarizes the results, which show the mean MIC and MBC values for each antibacterial agent tested. The increased antimicrobial activity of Ag-incorporated chitosan materials due to a high infiltration of the silver component results in a high bactericidal activity, which is consistent with our experimental findings results. These findings also show that chitosan-based antibacterial silver nano may have a dual mechanism of action. Chitosan is the enhanced activity as a result of Chi/Ag-NPs, which acts as a stabilizing agent as well as a carrier for Ag-NPs. This could also be due to the increased surface area and positive surface density, which allow for better interaction with negatively charged bacterial cell membranes, enhancing alteration in cell permeability and penetration of nano-sized particles into the bacterial cell, resulting in its death [59,60]. As expected, the mode of action could also be due to Ag+ interactions from the AgNPs and Chi/Ag-NPs conjugates with DNA or available proteins in the bacterial cell wall, which ultimately lead to cell death [61].

### 3.3. Anti-Biofilm Evaluation

**Figure 5:**
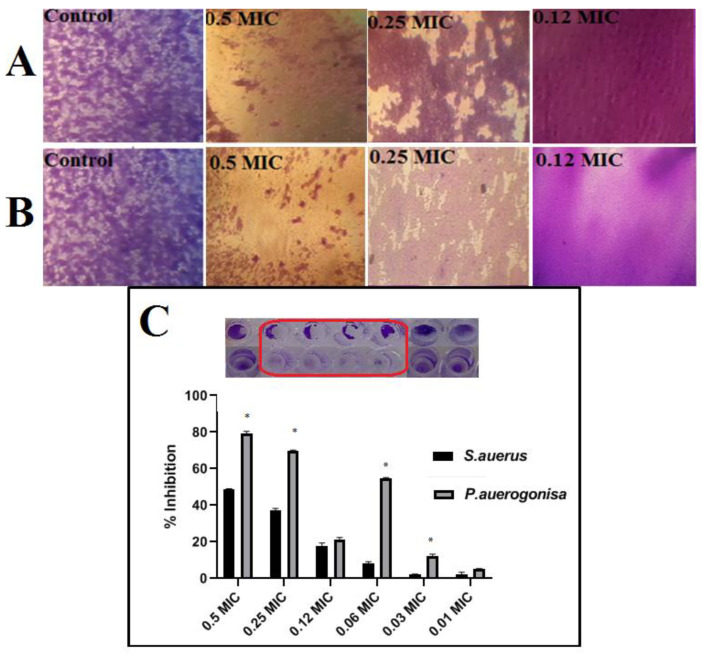
Light-inverted microscopic images of *S. aureus* (**A**) and *P. aeruginosa* (**B**) biofilms grown with various concentrations of Chi/Ag-NPs. At concentrations above 0.12xMIC bacteria have appeared as aggregated together to perform normal biofilm. *P. aeruginosa* and *S. auerus* biofilm inhibition in the presence of Chi/Ag-NPs at Sub. MIC (**C**). The absorbance of the control was considered to represent 100% of biofilm (results were considered significant when compared to control; * *p* < 0.05. Data are presented as mean ± SD, *n* = 4).

Biofilm inhibition was measured using a standard crystal violet assay, and the results were expressed as a percentage. Figure 5 shows the anti-biofilm activity of the Chi/Ag-NPs used in their synthesis at different concentrations. Bacterial biofilm inhibition of Chi/Ag-NPs demonstrated significant biofilm inhibiting activity (*p* < 0.05) against *P. aeruginosa* but not against *S. auerus*. In a biofilm quantification assay, test bacterial pathogens showed a concentration-dependent decrease in biofilm formation (Figure 5). The antibiofilm activity of Chi/Ag-NPs against *P. aeruginosa* at sub-MIC levels reduced biofilm formation by 78 and 69% at 0.5 × MIC and 0.25 × MIC, respectively. Furthermore, the antibiofilm activity of *S. auerus* by Chi/Ag-NPs at sub-MIC levels reduced biofilm formation by 48% and 36% at 0.5 × MIC, and 0.25 × MIC, respectively. At the sub-MIC levels tested, this compound had the highest inhibitory potential for biofilm formation against *P. aeruginosa* and *S. auerus* biofilm formation without inhibiting planktonic growth. One of the most important reasons for the effect of silver nanocomposites on biofilm inhibition activity is their particle size, as smaller particles have a larger surface area for interaction with microorganisms when compared to the bacterial control [62]. Chi/Ag-NPs hydrogel showed reduced biomass biofilm formation when compared to the bacterial control. Higher levels of biofilm reductase have been reported when AgNPs particles are less than 100 nm in size, which inhibits the synthesis and secretion of extracellular polysaccharides [63]. In the production and secretion of exopolysaccharides (EPSs) by bacterial cells, environmental signals trigger the production of EPS in bacteria. As a result, biofilm formation will be limited if EPS formation can be suppressed [64]. Our results indicated that Ag-NPs with a size of 9–65 nm in the Chi/Ag-NPs hydrogel help prevent biofilm formation. Biosynthesized AgNPs have previously been shown to have anti-biofilm activity against *P. aeruginosa* and *S. epidermidis* by AgNPs with a diameter of 100 nm, which reduced biofilm formation by 95–98% [62]. Another study reported on biofilm inhibition at 15 mg/mL from silver nanoparticles resulted in an 89% inhibition of biofilm in *S. aureus* and 75% in *E. coli.* The new findings also demonstrated that the bacteria studied are extremely sensitive to Chi/Ag-NPs, implying that the complicated biofilm signaling system is linked to cell viability. Recently, research has been undertaken on conjugating produced nanoparticles with polymers for use in combating biofilm development [65].

### 3.4. Antifungal Activity

**Figure 6:**
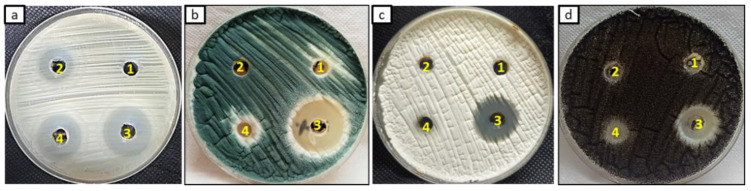
Antifungal activity of Chi (1), Ag+ (2), Chi/Ag-NPs (3), and Nystatin (4) toward *C. albicans* (**a**), *A. fumgatus* (**b**), *A. terreus* (**c**), and *A. niger* (**d**) using agar-well diffusion method.

In this study, the antifungal activity of Chi/Ag-NPs and starting materials was evaluated as shown in Figure 6 and Table 2. Results revealed that Chi/Ag-NPs exhibited promising antifungal activity against tested fungal strains compared to starting materials and nystatin as a standard antifungal. Inhibition zones of Chi/Ag-NPs towards *C. albicans, A. fumigatus, A. terreus,* and *A. niger* were 22, 29, 20, and 17 mm, respectively. On the other hand, start materials (Ag+ and Chi) exhibited very weak antifungal activity on some tested fungal strains. Nystatin exhibited promising antifungal activity against *C. albican* but gave weak antifungal against *A. fumigatus, A. terreus,* and *A. niger*. Therefore, the prepared Chi/Ag-NPs are promising as antifungal agents compared to nystatin. Furthermore, the MIC of Chi/Ag-NPs and starting materials were detected as shown in Table 2. Results illustrated that MIC_s_ of Chi/Ag-NPs against *C. albicans, A. fumigatus, A. terreus,* and *A. niger* were 250, 62.5, 500, and 1000 µg/mL, respectively. A Chi/Ag-NPs solution can be used as a bamboo-straw-coating material to inhibit the growth of *A. flavus* [66]. The outstanding antifungal activity of Chi/Ag-NPs is attributed to the positive charge of the amino group in chitosan is combined with negative charge components of the fungal cell, thus Chi/Ag-NPs may suppress the fungal growth by chelating various transitional metal ions, inhibiting enzymes and by impairing the exchange with the medium. The increase in the antimicrobial activity is due to the greater stability of Chi/Ag-NPs in an aqueous medium because chitosan protects them from aggregation [67].

### 3.5. Antioxidant Activity

**Figure 7:**
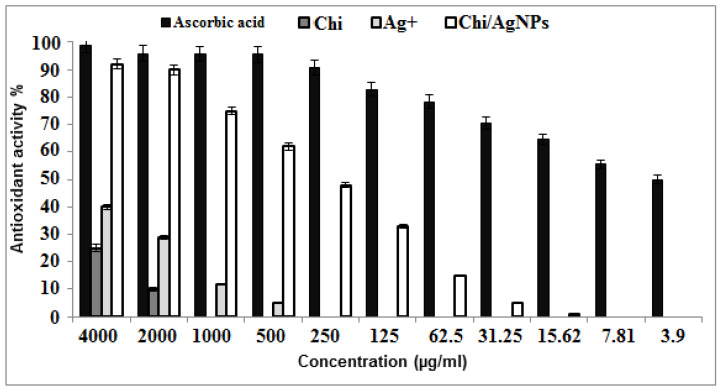
Antioxidant activity of Chi, Ag+, and Chi/Ag-NPs.

In biological systems, free radicals are generated as a result of the interaction of biomolecules with molecular oxygen [68]. Therefore, antioxidant compounds are used to resist the ROS effect. Antioxidant activity of Chi/Ag-NPs was evaluated compared to starting materials and ascorbic acid, as shown in Figure 7. The result showed that Chi/Ag-NPs revealed antioxidant activity were 92, 90, and 75% at concentrations of 4000, 2000, and 1000 µg/mL. Moreover, the IC_50_ of Chi/Ag-NPs was 261 µg/mL but was 3.9 µg/mL for ascorbic acid. The mechanism of action of the antioxidant activity of Chi/Ag-NPs is attributed to the binding of transition metal ion catalysts, decomposition of peroxides, inhibition of chain reaction, and inhibition of continued hydrogen abstraction [69]. The highest antioxidant activity is attributed to the presence of various bio-reductive groups of the phytochemicals present on the surface of the Ag-NPs [70].

### 3.6. Cytotoxicity on Normal Human Skin Cell Line (BJ-1)

**Figure 8:**
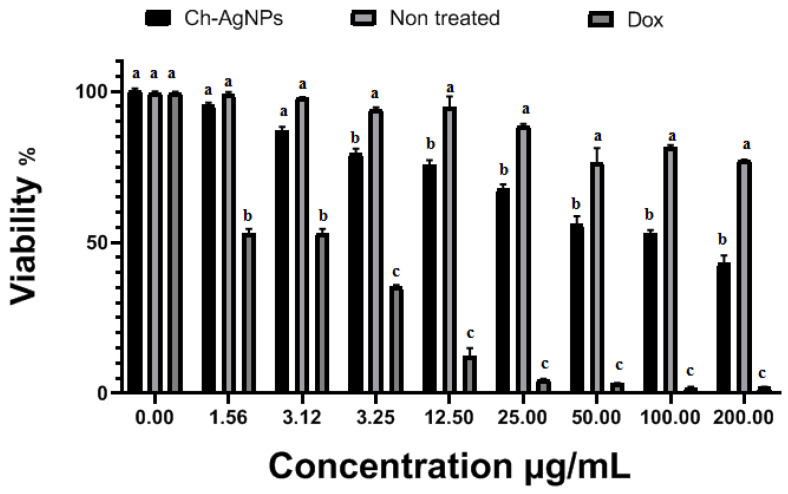
In vitro cytotoxicity effects on Chi/Ag-NPs and doxorubicin against normal human skin cell line (BJ-1) was assessed by SRB colorimetric assay. Within each column, different letters indicate significant differences among values (*p* < 0.05) based on one-way ANOVA estimated by GraphPad Instat software, (version 6.01), San Diego, CA, USA.

Cytotoxic studies are needed to avoid toxicity on normal cells. The in vitro cytotoxicity effects of Chi/Ag-NPs against normal human skin cell line (BJ-1) significantly inhibited the proliferation of (BJ-1) human skin cell line in a concentration-dependent manner (0–200 µg/mL). The half-maximal inhibitory concentrations (IC_50_) of Chi/Ag-NPs and Dox were calculated to be 119.2 and 3.9 µg/mL cells, respectively. So, according to the Special Programme for Research and Training in Tropical Diseases (WHO—Tropical Diseases), Chi/Ag-NPs were considered non-toxic (IC50 > 90.00 µg/mL) Figure 8. The toxicity of silver nanoparticles loaded with chitosan depends on concentration, species, and particle size [71]. In the present study, As previously reported by researchers [72], an increase in nanoparticle concentration increases the cytotoxic effect on normal cell lines in a dose-dependent manner. The half-maximal inhibitory concentrations (IC_50_) of Chi/Ag-NPs were calculated to be 119.2 µg/mL. Our results demonstrate that Chi/Ag-NPs are less toxic to normal human skin cell line (BJ-1) cells, whereas doxorubicin is more toxic.

### 3.7. Cell Migration Assay (Wound Scratch Assay)

**Figure 9:**
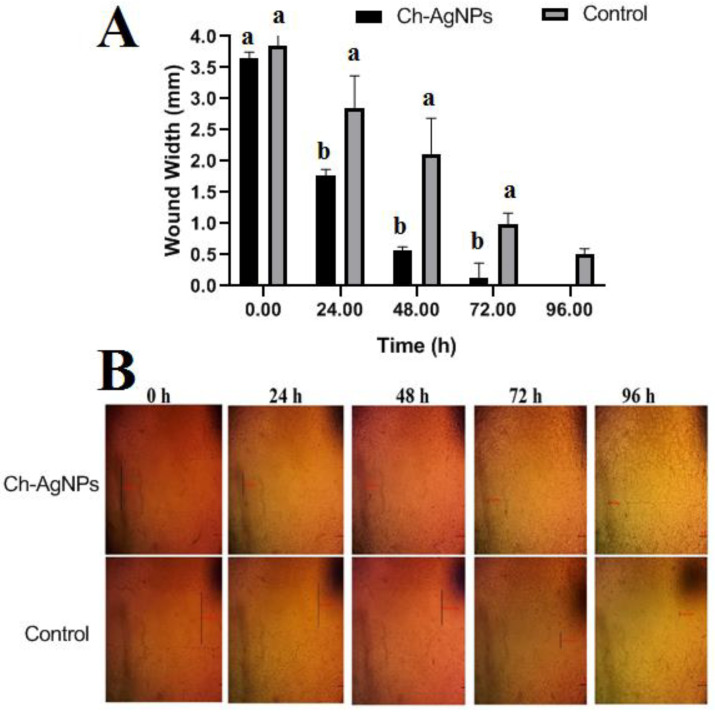
(**A**) Effects of different treatments on the wound area contraction (0–96 h). Values are given as mean ± SD (*n* = 3/group). Different letters indicate significant differences (*p* < 0.05). (**B**) Representative phase contrast micrographs of cells treated with 100 μg/mL Chi/Ag-NPs at 0 and 24 h. Wound closure rates are expressed as percentage of scratch closure from after 0 to 96 h compared to initial area. Red and black lines mean center and wide of the wound.

In this study, concentrations of Chi/Ag-NPs less than 100 µg/mL were used to investigate wound healing activities in human skin fibroblasts. The effects of Chi/Ag-NPs on the healing process were studied using an in vitro scratch wound healing assay. In Figure 9, fibroblasts advanced toward the opening to close the scratch wound by roughly 50.5% after a 24-h exposure to Chi/Ag-NPs, greatly accelerating the wound healing process compared to the control 17.5%. Because of its non-toxicity, anti-inflammatory impact, biocompatibility, retention of fibroblast growth factors, and stimulation of human skin fibroblast activities, chitosan has been widely employed as a wound dressing material [73]. It stimulates cell adhesion and proliferation and helps in the organization of the extracellular matrix. The above results are in line with those of Hajji et al. [74], which showed that silver nanoparticles prepared with chitosan promote wound healing, reduce infection, and reduce the risk of silver absorption. Based on these findings, it can be concluded that Chi/Ag-NPs can significantly speed up wound healing. The findings are consistent with those published by Souto et al. [75], who found that a spongy bilayer dressing containing CS–Ag nanoparticles dramatically expedited the healing of cutaneous wounds.

This section may be divided by subheadings. It should provide a concise and precise description of the experimental results, their interpretation, as well as the experimental conclusions that can be drawn.

## 4. Conclusions

In the current study, Chi/Ag-NPs with multifunctional biological purposes were prepared. Chi/Ag-NPs had highly antibacterial activity against gram-positive and gram-negative bacteria. They also reported antibiofilm activity as well as antioxidant activity. Furthermore, Chi/Ag-NPs exhibited promising antifungal activity towards unicellular and multicellular fungi. Chi/Ag-NPs were seen to significantly speed up wound healing at non-toxic concentrations due to their biocompatibility and good absorption of wound exudates. Data demonstrated the potentialities of Chi/Ag-NPs to be used as an alternative to antimicrobial drugs.

## Figures and Tables

**Table 1:** Minimum inhibition concentrations and minimum bactericidal concentrations of Chi, Ag+, and Chi/Ag-NPs samples against gram-negative *P. aeruginosa* and gram-positive *S. aureus*.

	MIC µg/mL		MBC µg/mL	
Ag+	25.0	50.0	100.0	ND
Chi	50.0	100.0	100.0	ND
Chi/AgNPs	12.5	12.5	25.0	50.0

ND: not detected; MIC: minimum inhibitory concentration, MBC: minimum bactericidal concentration.

**Table 2:** Inhibition zones and MIC of Chi/Ag-NPs compared to start materials.

Organisms	*C. albicans*		*A. fumgatus*		*A. terreus*		*A. niger*	
Materials	IZ/mm (4000 µg/mL)	MIC µg/mL	IZ/mm (4000 µg/mL)	MIC µg/mL	IZ/mm (4000 µg/mL)	MIC µg/mL	IZ/mm (4000 µg/mL)	MIC µg/mL
Chi	Nil	ND	8.00	4000	Nil	ND	Nil	ND
Ag+	16.00	1000	Nil	ND	Nil	ND	8.00	4000
Chi/Ag-NPs	22.00	250	29.00	62.5	20.00	500	17.00	1000
NS	21.00	250	10.00	2000	8.00	4000	9.00	4000

## Data Availability

Not applicable.
